# Trait-Associated SNPs Are More Likely to Be eQTLs: Annotation to Enhance Discovery from GWAS

**DOI:** 10.1371/journal.pgen.1000888

**Published:** 2010-04-01

**Authors:** Dan L. Nicolae, Eric Gamazon, Wei Zhang, Shiwei Duan, M. Eileen Dolan, Nancy J. Cox

**Affiliations:** 1 Department of Medicine, University of Chicago, Chicago, Illinois, United States of America; 2 Department of Human Genetics, University of Chicago, Chicago, Illinois, United States of America; 3 Department of Statistics, University of Chicago, Chicago, Illinois, United States of America; Georgia Institute of Technology, United States of America

## Abstract

Although genome-wide association studies (GWAS) of complex traits have yielded more reproducible associations than had been discovered using any other approach, the loci characterized to date do not account for much of the heritability to such traits and, in general, have not led to improved understanding of the biology underlying complex phenotypes. Using a web site we developed to serve results of expression quantitative trait locus (eQTL) studies in lymphoblastoid cell lines from HapMap samples (http://www.scandb.org), we show that single nucleotide polymorphisms (SNPs) associated with complex traits (from http://www.genome.gov/gwastudies/) are significantly more likely to be eQTLs than minor-allele-frequency–matched SNPs chosen from high-throughput GWAS platforms. These findings are robust across a range of thresholds for establishing eQTLs (p-values from 10^−4^–10^−8^), and a broad spectrum of human complex traits. Analyses of GWAS data from the Wellcome Trust studies confirm that annotating SNPs with a score reflecting the strength of the evidence that the SNP is an eQTL can improve the ability to discover true associations and clarify the nature of the mechanism driving the associations. Our results showing that trait-associated SNPs are more likely to be eQTLs and that application of this information can enhance discovery of trait-associated SNPs for complex phenotypes raise the possibility that we can utilize this information both to increase the heritability explained by identifiable genetic factors and to gain a better understanding of the biology underlying complex traits.

## Author Summary

We show here that single nucleotide polymorphisms (SNPs) associated with complex traits (as identified in the catalog of results from genome-wide association studies http://www.genome.gov/gwastudies/) are more likely than other SNPs chosen from high-throughput genotyping platforms to predict expression levels of genes. These observations confirm that genetic risk factors for complex traits will often affect phenotype by altering the amount or timing of protein production, rather than by changing the type of protein produced. This knowledge can be used to improve our ability to discover genetic risk factors for complex traits and to improve our understanding of their underlying biology.

## Introduction

Results of genome-wide association studies (GWAS) in complex traits published to date have provided us with surprisingly little new information on the nature of the genetic component to these phenotypes, despite the large number of single nucleotide polymorphisms (SNPs) found to be reproducibly associated with such traits. In some cases, this reflects the fact that major aspects of the biological basis for disease were already well understood; results of GWAS in autoimmune disorders, for example, have reinforced the central importance of the immune system and its regulation. For a few disorders, results of GWAS have highlighted biological contributing factors to disease that had not previously been recognized as central, such as the complement system in macular degeneration [1] or autophagy in Crohn's disease [2]–[4]. While it does seem ungrateful to question the utility of GWAS when they have yielded so many more reproducible associations than we have achieved with any other approach, the fact is that our primary goal in conducting GWAS for a complex trait – achieving a comprehensive understanding of the genetic basis for that trait – remains elusive for most of the traits that have been examined. The failure to achieve this goal is made particularly acute by the recognition that the loci that have been successfully identified not only provide us with little insight into the genetic basis for the trait, but also account for little of the overall heritability [5]. Although there are important caveats to these grim statistics – most published studies have conducted discovery research in populations of European descent, and some important disorders have not even been examined using GWAS yet – the collective experience has led to sharp disagreement as to whether there is sufficient value in targeting near-term investments in genomics to GWAS, or whether such investments are better targeted to sequencing [6]–[8]. The key issue in this controversy is whether the genetic risk factors not yet discovered are largely similar in frequency and effect size to those that have been discovered using GWAS (i.e. common alleles with low risk) or are, instead, rarer alleles that would be best identified through sequencing studies.

Studies we report here suggest that we have not yet exhausted the signals that can be discovered through GWAS. Annotating SNPs with information on expression can improve our ability to more easily distinguish those associations likely to be replicated, and provide us with a better understanding of the genes and mechanisms driving the associations we discover. Moreover, it appears that for at least a subset of complex disorders, there are many more common variants truly associated with disease and highly likely to be expression quantitative trait loci (eQTLs). These variants can be identified and characterized using existing GWAS and tools such as the SCAN database (SNP and Copy number ANnotation; http://www.scandb.org) [9].

## Results

### SNPs Associated with Complex Traits Are More Likely to Be eQTLs than Frequency-Matched SNPs from GWAS Platforms

**Figure 1:**
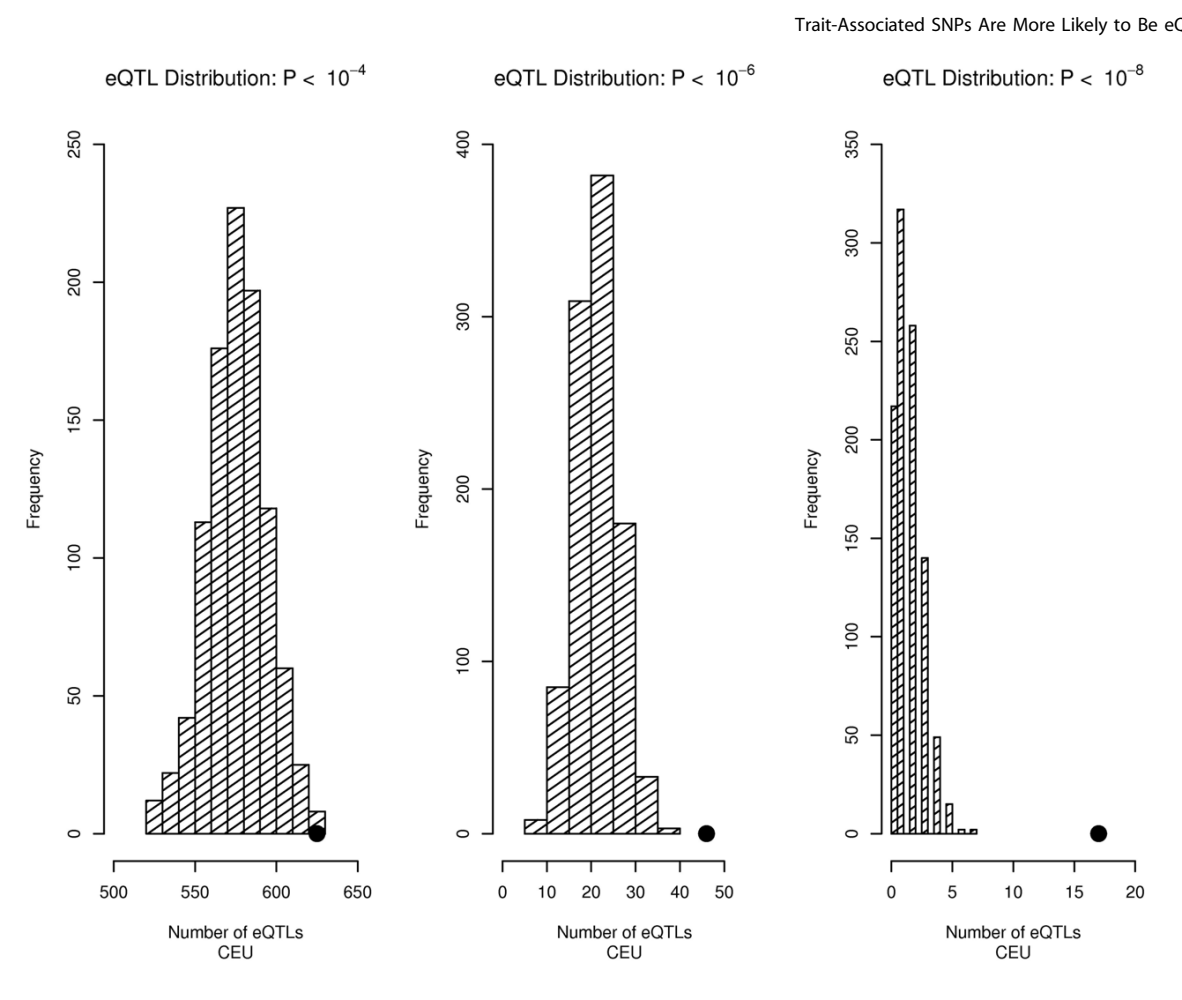
Trait-associated SNPs are more likely to be eQTLs. The distribution of the number of eQTLs (defined as p<10^−4^ left panel, p<10^−6^ middle panel, and p<10^−8^ right panel) observed for each of 1,000 draws of 1,598 SNPs from bins matched for minor allele frequency to the 1,598 SNPs downloaded from the NHGRI catalog (bins include all SNPs in the Illumina 1M and Affymetrix 6.0 products) is shown in the bar graphs, with the actual number of eQTLs observed in the 1,598 SNPs from the NHGRI catalog shown as a solid circle.

The 1598 SNPs characterized in the GWAS catalog [10] as showing association with complex traits included 625 that would be classified as eQTLs with a p-value threshold of 10^−4^ using CEPH (Centre d'Etude du Polymorphisme Humain) samples of European descent from Utah (CEU) or 972 using the combined sample of the CEU plus the Yoruban samples from Ibadan Nigeria (YRI) data to define eQTLs, 46 (83 for the combined CEU+YRI) that would be classified as eQTLs with a p-value threshold of 10^−6^ and 17 (18 for the combined CEU+YRI) that would be classified as eQTLs with a p-value threshold of 10^−8^. As summarized in Figure 1, we observed significantly more eQTLs among these 1598 trait-associated SNPs than expected given their minor allele frequency (MAF) distribution. Given that many gene transcript levels show substantial correlations, it is, perhaps, not surprising that we also observed that SNPs associated with many transcripts (sometimes called eQTL hot spots or master regulators) were also enriched among trait-associated SNPs. Using a more stringent definition for defining trait-associated SNPs (up to 10^−8^) reduced their absolute numbers of such SNPs, but increased the significance of the observed enrichment of eQTLs among trait-associated SNPs.

There was some redundancy among the set of SNPs examined through the GWAS catalog because different published reports can highlight different (but correlated) SNPs for the same phenotypes, and also because several phenotypes may show associations to some of the same sets of SNPs (e.g. autoimmune disorders with associations to HLA region SNPs and *PTPN22*). To insure that our conclusions were robust to both linkage disequilibrium (LD) among the cataloged SNP set and which of any correlated set of SNPs were retained for analyses, we conducted simulations in which we retained only one of any SNP set with r^2^>0.3 and for each replicate conducted 100 simulations conditional on MAF as for Figure 1 (see Materials and Methods for simulation details). The eQTL enrichment was preserved in LD-pruned sets of cataloged variants, for eQTL definition thresholds with p<10^−4^ or smaller.

Table 1 summarizes the information on eQTLs for SNPs (with MAF>0.05) on high throughput platforms, associated with traits in the GWAS catalog, and associated with phenotypes selected to represent those for which lymphoblastoid cell lines (LCLs) might be expected to be a poor proxy for the human tissues most relevant for disease (neurological/psychiatric phenotypes, cancers) and those for which LCLs might be expected to be a good proxy for the human tissues most relevant to disease (autoimmune disorders). As is apparent from the table, neurological and psychiatric phenotypes as well as cancers (regardless of tumor site) showed levels of enrichment similar to those observed for the overall trait-associated SNP set, although as expected, autoimmune disorders showed somewhat greater levels of enrichment. Division of the GWAS catalog SNPs into those identified through studies on autoimmune disorders and those identified through studies on other complex traits reveals that the apparent enrichment of eQTLs (defined at a threshold of p<10^−6^) detected through studies on LCLs is indeed significantly greater (p = 0.00011) for SNPs identified through studies in autoimmune disorders than for SNPs identified through studies of other traits.

**Table 1:** Number (proportion) of SNPs classified as eQTLs for diseases with different focal tissues.

Using eQTLs from CEU+YRI	# SNPs	eQTL p-value threshold 10^−4^	eQTL p-value threshold 10^−6^
Platform SNPs (MAF >.05)	1,213,906	595,285 (.490)	29,347 (.024)
All Catalog SNPs	1598	972 (0.608)	83 (0.052)
Autoimmune Disorders	259	165 (0.637)	21 (0.081)
Cancers	93	56 (0.602)	4 (0.043)
Neurological/Psychiatric Disorders	63	41 (0.651)	2 (0.032)
**Using eQTLs from CEU only**			
Platform SNPs (MAF >.05)	1,213,906	345,249 (.284)	12,749 (.011)
All Catalog SNPs	1598	625 (0.391)	46 (0.029)
Autoimmune Disorders	259	116 (0.448)	17 (0.066)
Cancers	93	30 (0.323)	3 (.032)
Neurological/Psychiatric Disorders	63	20 (0.317)	1 (0.016)

SNPs from the NHGRI catalog were classified according to the type of disease leading to their inclusion as a trait-associated SNP to investigate whether eQTLs identified in LCLs are more enriched in disorders for which LCLs are more likely to be an appropriate tissue match for the disease. Studies were conducted separately using eQTLs identified in the combined CEU+YRI samples (upper lines), and those identified only in the CEU (lower lines). Only SNPs with MAF >0.05 are included in these studies, and eQTL p-value thresholds of 10^−4^ and 10^−6^ are shown.

### Using eQTL Information Improves Ability to Identify Reproducible Signals in GWAS

**Figure 2:**
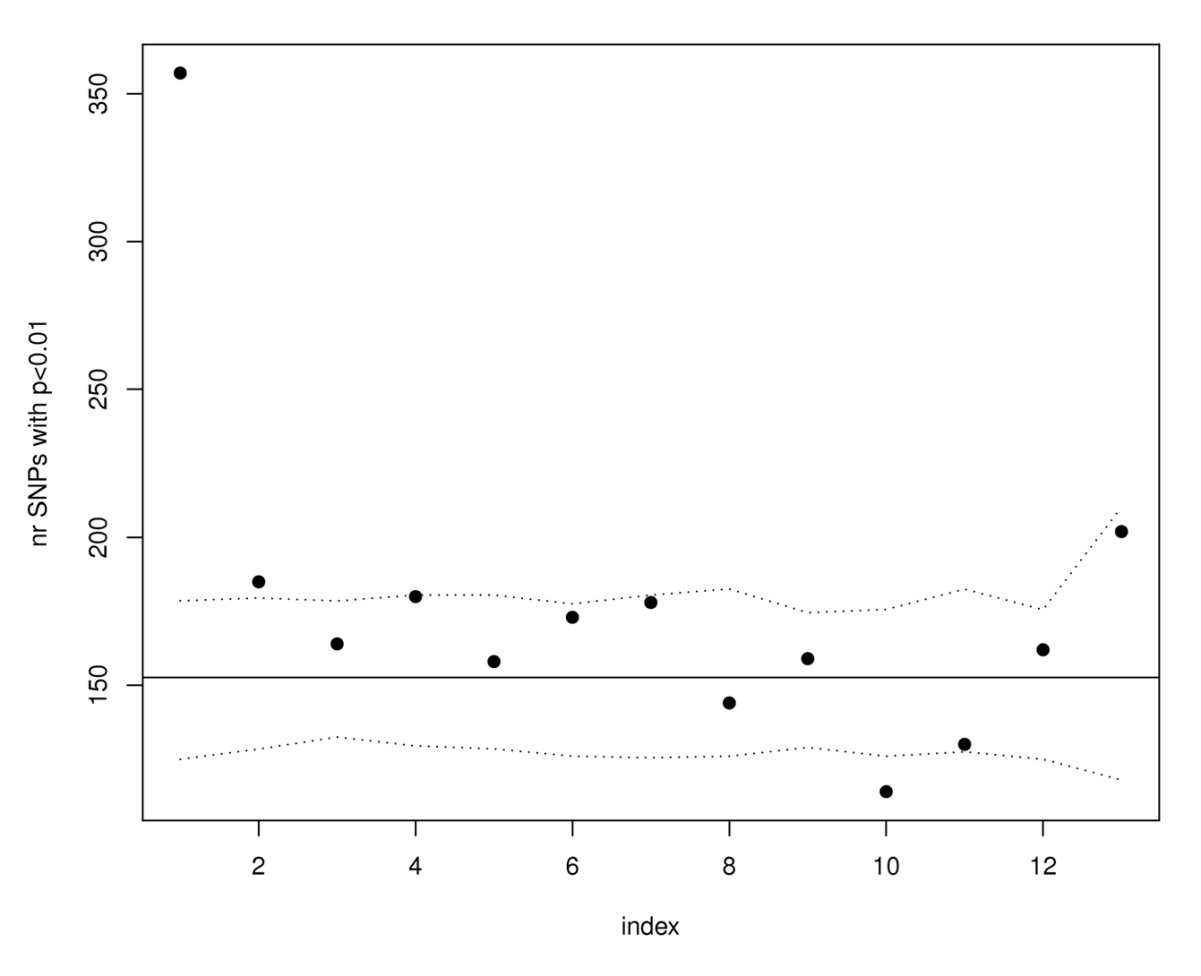
SNPs with the highest eQTL function scores are enriched for WTCCC Crohn's susceptibility loci. SNPs were ordered according to their eQTL function scores from most significant to least significant and divided in groups of 10,000. Only the data from the first 13 groups is shown (as 13 index bins along the X-axis) because fewer than 130K SNPs have expression scores larger than zero. Each point in the graph corresponds to eQTL function score bins and the y-axis shows the number of SNPs in each bin that have WTCCC Crohn's GWAS p-values less than 0.01. The horizontal line illustrates the expectation based on the observed number of SNPs in the whole GWAS that have p-values smaller than 0.01. The proportion of the remaining SNPs with p smaller than 0.01 is 0.014. The dotted lines represent estimated 95% confidence bands obtained using simulations.

In order to understand the practical utility of our observation that trait-associated SNPs are more likely to be eQTLs, we examined in more detail results summaries from the Wellcome Trust Case Control Consortium (WTCCC) GWAS [11], using the data on Crohn's disease as a primary example application. If SNPs associated with Crohn's disease are no more likely than non-associated SNPs to be eQTLs, we would expect the proportion of SNPs associated with Crohn's disease at p<.01 in bins defined according to eQTL function score (see Materials and Methods for definition of eQTL function score) to be constant (the overall proportion of Crohn's p-values smaller than .01 is .0152). Instead, as illustrated in Figure 2, we found that there were 357 SNPs associated with Crohn's disease among the 10,000 SNPs with the highest eQTL function scores; the expected number, based on 100 simulations conditioned on MAF for these 10,000 SNPs was 117–178. No such enrichment was observed for other ways of prioritizing SNPs for function, including missense SNPs (the proportion of non-synonymous SNPs with Crohn's association p<0.01 was 0.0152), coding-synonymous substitutions (0.0253), or intronic SNPs (0.0158). We then differentiated the SNPs as “cis-regulators” (these are eQTLs for genes that are within 4 Mb) and “trans-regulators” (these are eQTLs for genes that are more than 4 Mb away or on a different chromosome). The Bonferroni adjustment of significance was different for the cis- and trans-regulators (see Materials and Methods). The enrichment was preserved in the SNPs classified as cis-regulators but was not evident in the SNPs classified as trans-regulators, suggesting that cis-regulatory effects were more likely to be present among the Crohn's associated SNPs.

**Figure 3:**
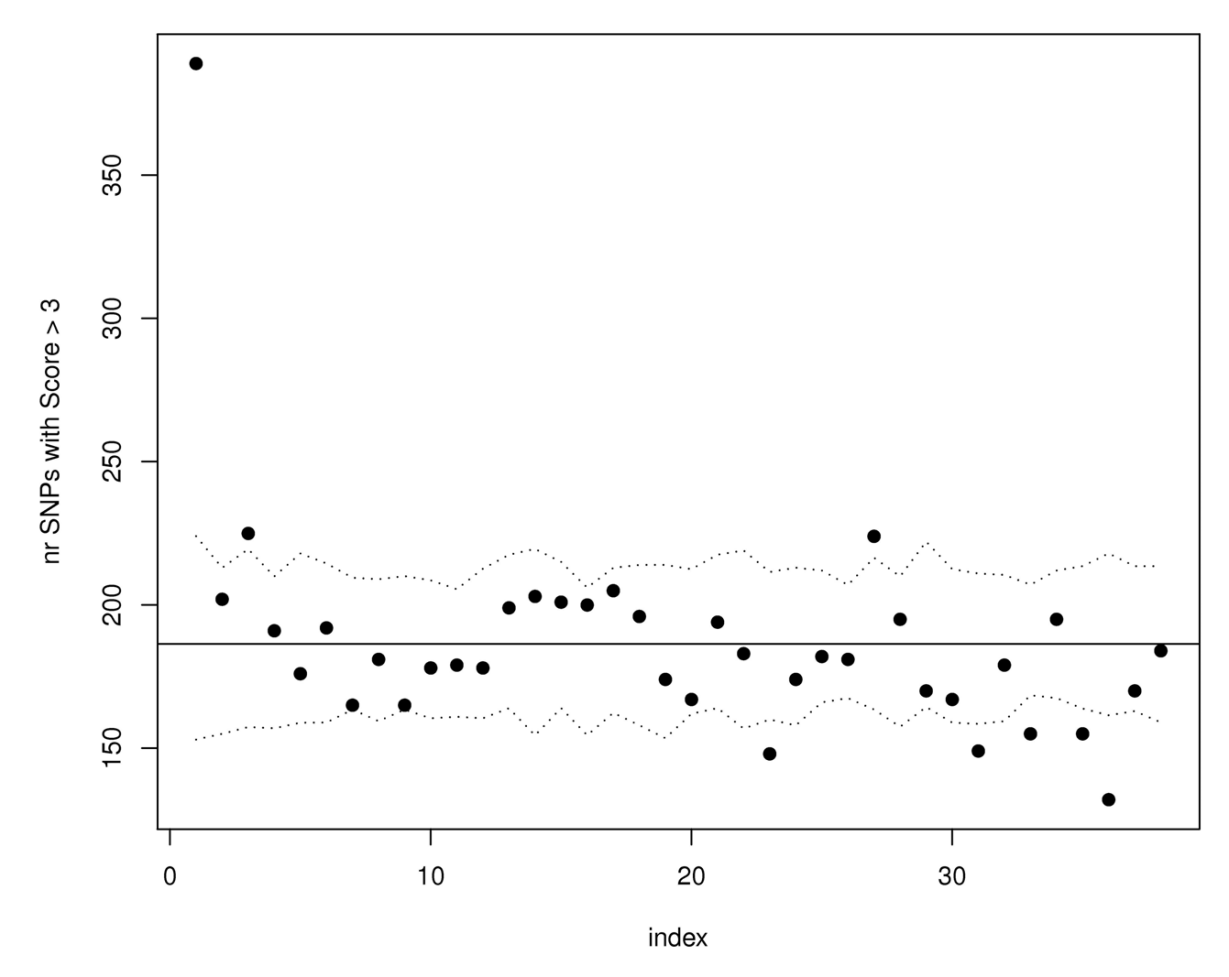
SNPs with the strongest evidence for association in WTCCC Crohn's disease are more likely to have eQTL function scores >3. SNPs have been ordered according to their WTCCC Crohn's association p-values from the most to the least significant, and divided in groups of 10,000. For each group bin of 10,000 trait-associated SNPs, the number of SNPs with expression score larger than 3 was calculated and the results are shown in the scatterplot. The horizontal line illustrates the expectation based on the observed eQTL function scores in all SNPs in the WTCCC Crohn's dataset. The dotted lines represent estimated 95% confidence bands obtained using simulations.

We observed a similar enrichment if we start with the SNPs most strongly associated with Crohn's disease and ask whether there was enrichment for SNPs with high eQTL function scores. As illustrated in Figure 3, in the top 1000 Crohn's associated SNPs we observed 392 with eQTL functional scores larger than 1 (the expected number based on all HapMap scores is 183.5), 324 SNPs with scores larger than 2 (143.5 expected by chance) and 172 with scores larger than 3 (with only 18.6 expected by chance). Among these 172 SNPs we observed some, such as in *STAT3*, that have been confirmed in replication studies.

**Figure 4:**
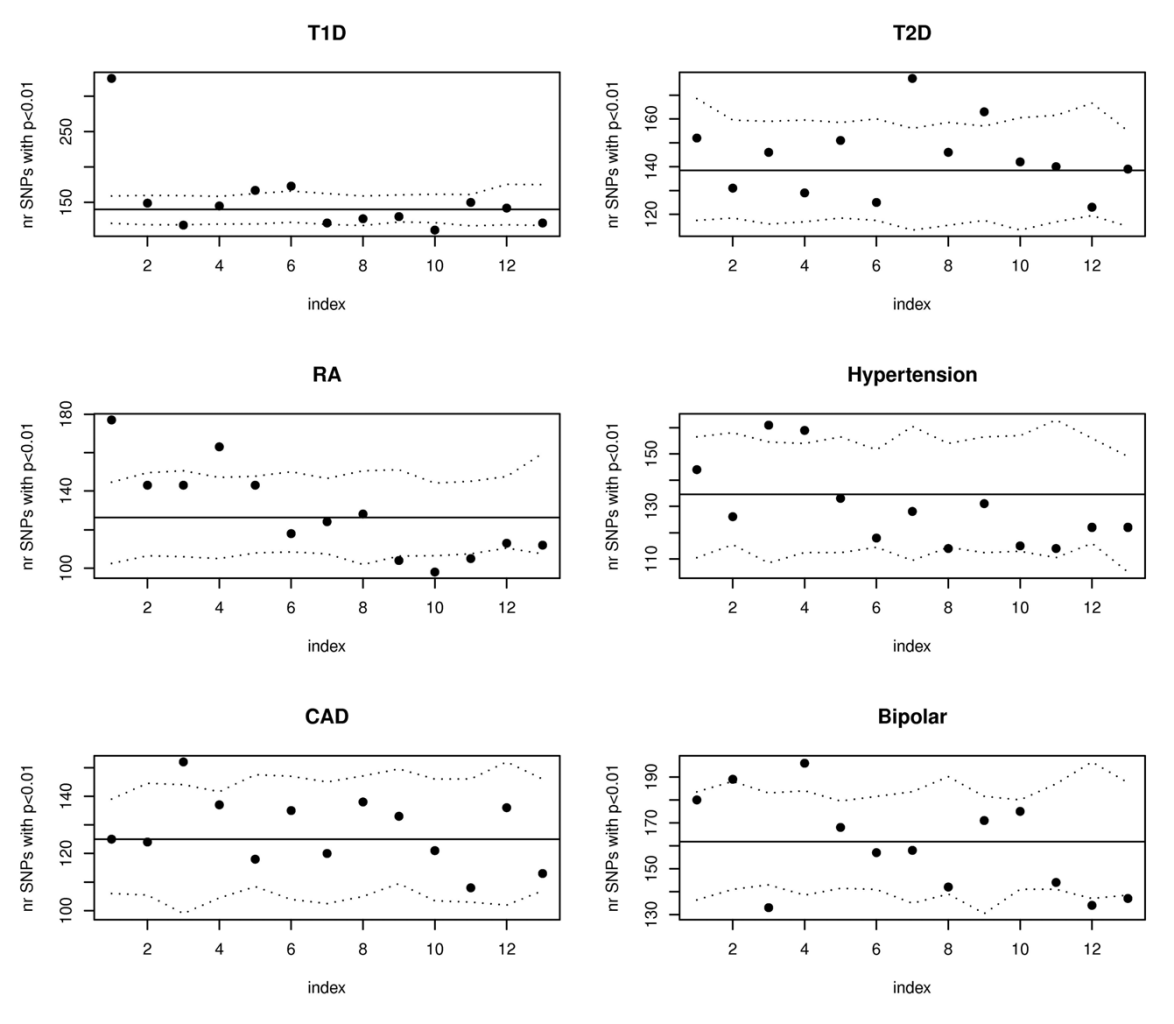
SNPs with the highest eQTL function scores are enriched for WTCCC T1D and RA susceptibility loci. The plots show results of eQTL enrichment analyses for the remaining Wellcome Trust phenotypes beginning with SNPs having the highest expression scores (similar to analyses for Crohn's disease summarized in Figure 2). SNPs have been ordered according to their expression score from most significant to least significant and divided in groups of 10,000. Only the data from the first 13 groups is shown. The six plots correspond to the six other diseases investigated in the initial WTCCC GWAS. The dotted lines represent estimated 95% confidence bands obtained using simulations.

**Figure 5:**
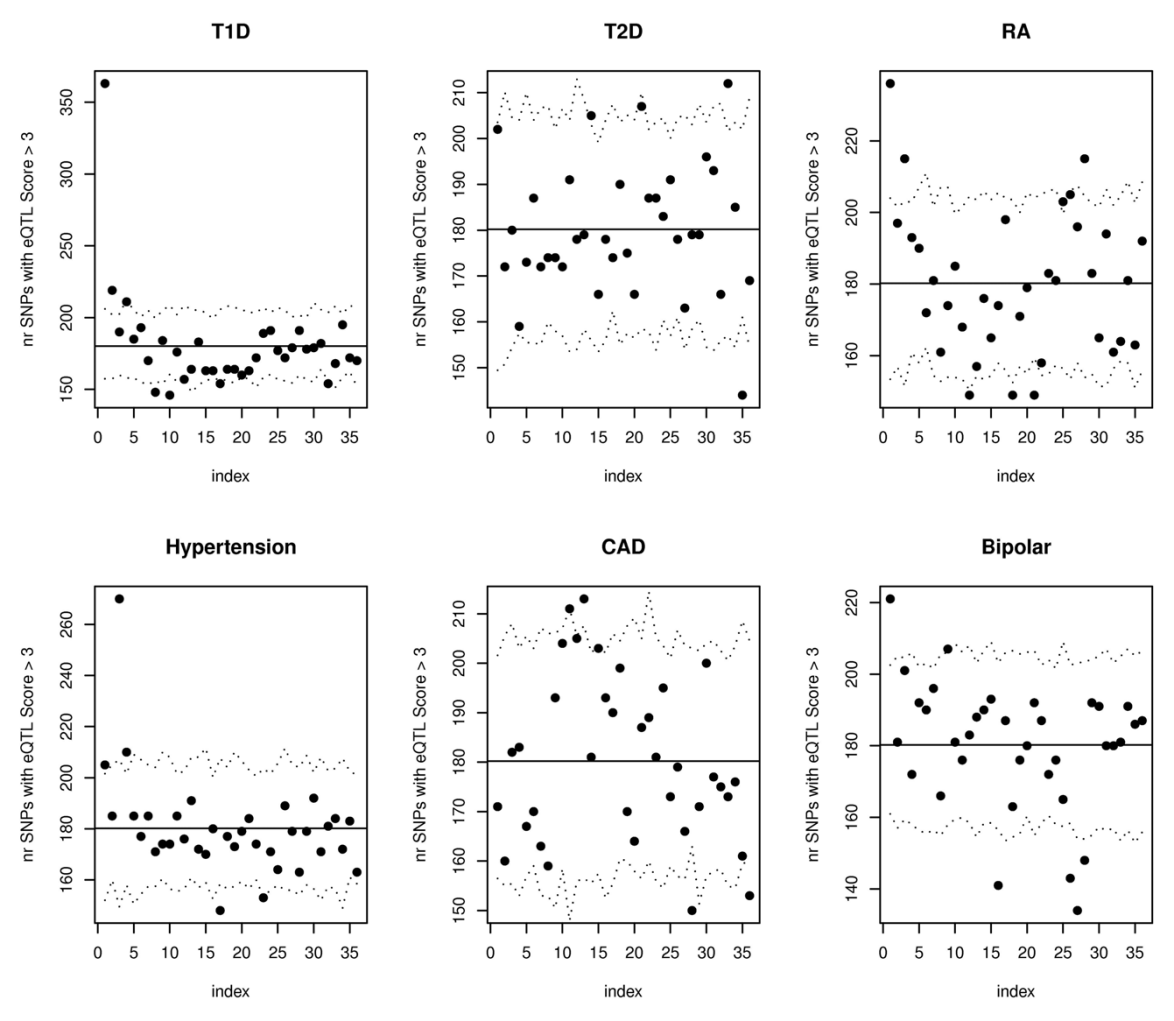
SNPs with the strongest evidence for association with WTCCC T1D, RA, hypertension, and bipolar disorder are more likely to have eQTL function scores >3. The plots show results of eQTL enrichment analysis for the remaining Wellcome Trust phenotypes beginning with the SNPs most strongly associated with disease (similar to analyses for Crohn's disease summarized in Figure 3). For each disease, SNPs have been ordered according to their association p-values from the most to the least significant, and divided in groups of 10,000. For each group, the number of SNPs with expression score larger than 3 was calculated and the results are shown in the scatterplots. The horizontal lines illustrate the expectation based on the observed scores in all SNPs in the relevant WTCCC dataset. The dotted lines represent estimated 95% confidence bands obtained using simulations.

Analyses on other phenotypes from the WTCCC association studies [11] confirmed that our proposed annotations benefit phenotypes beyond Crohn's disease. Figure 4 illustrates that both type 1 diabetes (T1D) and rheumatoid arthritis (RA) had significantly more SNPs than expected with phenotype associations (p<.01) among the 10,000 SNPs with top eQTL function scores. To insure that these results were not driven by strong LD among SNPs in the major histocompatibility complex (MHC) region, we repeated the analyses for all Wellcome Trust phenotypes after removing all SNPs on chromosome 6, with substantially similar results. Figure 5 highlights the enrichment of functional SNPs (eQTL function scores larger than 3) among the SNPs with the strongest associations to disease in the scans for T1D and RA (not unexpected given results in Figure 4), but also for hypertension and bipolar disease. There is little evidence for enrichment of eQTLs among top signals for coronary artery disease (CAD) or type 2 diabetes (T2D). These results suggest that for at least some complex traits, a more efficient prioritization for replication studies can be achieved by incorporating the expression scores, and that there may be many more SNPs that are truly associated with disease and also likely to be eQTLs than we have identified through GWAS to date.

## Discussion

Our results build on those of previous studies, particularly those of Schadt and colleagues [12]–[15] that focused on using transcriptome information to improve understanding of genetic signals, but the rapid accumulation of trait-associated SNPs through GWAS, coupled with the systematic efforts to catalog these variants [10],[16] has enabled us to generalize the concept that information on eQTLs will have utility for understanding the genetic component to complex traits. Our primary observations – that SNPs reproducibly associated with complex human traits are more likely to be eQTLs – are novel, although perhaps not unexpected given that few of even those SNPs most reproducibly associated with complex traits have been attributed to missense or nonsense variants. Our subsequent observations made in association studies in which we annotated SNPs with eQTL information, imply that at least some complex disorders have substantial numbers of undiscovered susceptibility loci that can be more easily discovered and characterized by annotating SNPs with information on eQTL scores. This is also a novel observation. Moreover, use of information on expression appears to benefit our understanding of not only more tractable disorders, such as Crohn's disease and autoimmune disorders, for which relatively large numbers of loci have already been identified at the cost of genotyping 10,000 or fewer samples, but also for less tractable disorders such as hypertension and bipolar disease, for which fewer associations have been characterized despite the large numbers of genotyped samples (>29,000 in GWAS and >34,000 for replication in studies of hypertension [17]).

Use of expression information should move us closer to that elusive goal of developing a more comprehensive understanding of the genetic basis for complex traits in two ways: (1) by identifying and characterizing a larger number of contributing loci, we should be better able to discern the key biological functions affected by genetic risk factors and (2) SNP signals may be more accurately characterized with respect to target genes and mechanism of effect, further enhancing our ability to discern relevant biological functions. Thus, our results provide a strong additional rationale for the inclusion of eQTL information in the annotation of SNPs from GWAS. Moreover, even the analyses we have completed to characterize trait-associated SNPs as being more likely than allele frequency matched SNPs from the same platform to be eQTLs have revealed information about the potential biological rationale for some of the observed associations. For example, an intronic SNP, rs3129934, on chromosome 6 that is a cis-acting eQTL for multiple HLA transcripts with a high eQTL functional score (24.9) shows the strongest association with T1D in the MHC region in the WTCCC study [11] and is also the SNP most strongly associated with increased risk for multiple sclerosis (MS) in a pooling-based GWAS [18].

A variety of the approaches we used in conducting these studies should be of general utility in enrichment studies. It is clear, for example, that the MAF spectrum of SNPs associated with complex human traits is quite different than that of SNPs on the products used to conduct GWAS, or in the overall HapMap. The much higher minor allele frequencies observed for SNPs reproducibly associated with complex traits than for SNPs on high throughput platforms is a natural consequence of the increased power to detect associations for SNPs with higher minor allele frequencies. It is clearly critical to condition on the MAF in enrichment studies in which simulations are conducted using SNPs from high throughput platforms or from the HapMap, and to date, this has not been widely appreciated. The correlation among SNPs due to LD is a further complicating factor in conducting enrichment studies. We are confident that failure to adequately account for LD will not affect our estimate of the proportion of SNPs that are eQTLs, but rather the variance of this estimate. There are, however, more subtle biases related to LD that might have affected our studies. For example, true associations may be more easily detected in regions of the genome with high LD because these regions are more likely to have good coverage on high throughput platforms for GWAS. Although it is unclear that this would bias results of our studies, it is important to note that these types of subtle biases can affect results of enrichment studies in unpredictable ways.

The challenges of conducting eQTL studies are also widely appreciated. The eQTLs identified in array-based transcriptome studies in LCLs are likely to be a limited subset of the eQTLs that will ultimately be identified using more sensitive techniques in comprehensive collections of human tissues. Moreover, a variety of studies have highlighted the potential sensitivity of eQTL studies to cell and tissue subtypes [15],[19],[20]. Although several studies have suggested that eQTLs will be largely tissue specific [21],[22], others note that substantial numbers of eQTLs are shared across tissues [15],[23]. Similarly, some have found that LCLs are prone to such high levels of non-genetic variability (growth parameters, EBV levels, ATP levels, etc.) that they might be considered unreliable for genomic studies [24], while others highlight the reproducibility of results of transcriptome studies [25],[26] and find that LCLs show little variability as a consequence of non-genetic factors [27].

To the degree that the transcriptome is a reflection of the local and temporal environment, it could be argued that eQTLs identified in generic studies will have little value for enhancing understanding of complex trait studies unless eQTLs are identified in relevant tissues of the individuals participating in the complex trait studies. Our results provide little support for such arguments, and instead highlight the utility of LCLs as a reasonable model for the study of expression. And while LCLs are clearly not the ideal tissue for expression studies in many complex disorders, we note that we plan to serve results of eQTL studies in a variety of tissues in SCAN, and consider the studies summarized in this manuscript to be an initial effort that will be enriched by subsequent studies in additional tissues. It is possible that diseases without evidence for eQTL enrichment using data from LCLs will ultimately yield such evidence when data from more relevant tissues is used; alternatively, at least a subset of those disorders without evidence for enrichment for eQTLs may turn out to derive substantial heritability from rare variants. In any case, results of our studies clearly support additional investment in eQTL identification in other tissues, as well as in the development of public access databases and software tools, such as those in SCAN (http://www.scandb.org), that allows results of such studies to be productively used by the scientific community.

## Materials and Methods

### SNPs Reproducibly Associated with Complex Human Traits

The National Human Genome Research Institute (NHGRI) has collected the results of published GWA studies in a publicly available online database (http://www.genome.gov/gwastudies) [16]. This catalog is continually updated; the version we utilize for the present study was downloaded on June 29, 2009. We used the NHGRI default set of SNPs reported to be associated to complex traits with a p-value of at least 10^−5^ for all figures and tables in the manuscript, but we note that results show more significant enrichment for more stringent definitions for trait-associated SNPs (we tested 10^−5^ through 10^−8^).

### The SCAN Database

We have created an online database to serve the results of our gene-expression studies in LCLs. SCAN (SNP and Copy number ANnotation database; http://www.scandb.org) can be queried through either the SNP or gene to retrieve information on the relationship between SNPs and transcript levels at user-specified p-value thresholds [9]. SCAN enables batch queries of genes to retrieve a list of SNPs that predict the expression of the genes at a user-specified threshold. SCAN also holds multi-locus disequilibrium measures [28] to summarize reported LD information among SNPs and to characterize coverage of genes by the high-throughput genotyping platforms.

The expression data served in SCAN had been assayed in HapMap LCLs (87 CEU and 89 YRI) with the Affymetrix GeneChip Human Exon 1.0 ST Array [29]. The exon array measures both exon-level and gene-level expression, includes approximately 1.4 million probesets, and profiles over 17,000 gene transcripts.

Genome-wide association analyses of 13,080 transcript clusters (gene-level) with reliable expression and more than 2 million SNPs in the CEU or YRI populations were conducted using the Quantitative Trait Disequilibrium Test (QTDT) [30]. A transcript cluster or probeset in the exon array is defined as reliably expressed in LCLs if the log_2_-transformed expression signal is greater than 6 in at least 80% of the 176 HapMap samples included analyses. Each transcript cluster includes a set of probesets (exon-level) containing all known exons and 5′- and 3′- untranslated regions (UTRs) in the genome. In contrast to other arrays (e.g., U95 and U133 series arrays), the probes on the exon array cover entire gene regions and not just 3′-UTRs.

Since SNPs in probes can generate spurious eQTL signals by affecting hybridization, we downloaded SNP data from dbSNP (build 129, human genome assembly build 36) to identify probes in probesets that hybridize to regions containing SNPs. The genomic coordinates of the probes were downloaded from the Affymetrix website (http://www.affymetrix.com). We filtered these probes before conducting the expression analyses generating the results served in SCAN.

### Analysis of eQTL Enrichment

All analyses have been conducted using all available information (eQTLs for both CEU and YRI samples) and also using only information from the CEU samples. Results are substantially similar, but because most GWAS reported to date have been conducted in samples of recent European descent, and to be conservative, summary figures use only the eQTL information from the CEU samples. We also provide details for some of the key eQTL studies in tabular form (Table 1), where we provide summary information for enrichment studies using CEU+YRI eQTL data and for enrichment studies using only the CEU eQTL data.

The MAF distribution for SNPs showing reproducible associations with complex human traits is markedly different from the MAF distribution for all SNPs on the high density SNP genotyping platforms, or the minor allele frequency distribution of SNPs included on high throughput platforms used for GWAS (Figure S1). Thus, any study focused on enrichments among SNPs reproducibly associated with complex human phenotypes must be conditioned on MAF. To enable us to conduct simulations conditional on MAF, we constructed MAF bins as follows: allele frequencies were calculated from HapMap genotype data on the CEU. Only the parents' genotypes were included in the frequency calculations since the children's alleles are not independent. We used the toolkit PLINK [31] to calculate frequencies. We classified SNPs on Affymetrix Genome-Wide Human SNP Array 6.0 and Illumina's High Density Human 1M-Duo into non-overlapping minor allele frequency (MAF) bins, each of width .05, using the MAFs of the SNPs in the CEU samples, as most of the published reports summarized in the NHGRI catalog are from GWAS on individuals of recent European descent.

We conducted simulations to test for an enrichment of eQTLs among the NHGRI variants associated with complex traits by generating 1000 randomized SNP sets *S_i_* each of the same size as the original NHGRI list (1598) containing variants matched on MAF distribution, sampled without replacement from the set of typed SNPs on each high-throughput platform, which had been grouped into discrete MAF bins. For each set, we determined the number of eQTLs, *Q_i_*, at a p-value threshold. We tested the robustness of the eQTL enrichment across a range of p-value thresholds (10^−8^ to 10^−4^), as shown in Figure 1. These simulations (N = 1000) yield an empirical p-value, calculated as the proportion of simulations in which the number of eQTLs exceeds the observed number, *Q*, in the NHGRI list.

Because some GWAS report associations for SNPs that were interrogated through imputation rather than direct genotyping, we repeated the analysis described above except that we used the entire set of HapMap SNPs to generate the 1000 randomized SNP sets with MAF matched to catalog SNPs, rather than only the SNPs included on high throughput GWAS platforms. Results of these studies show an even more significant enrichment of eQTLs among catalog SNPs (Figure S2). Thus, we focus in this paper on the more conservative results obtained using the simulations generated from SNPs on high-throughput GWAS platforms.

To investigate whether the eQTL enrichment we observe in the NHGRI variants is driven by linkage disequilibrium (LD), an LD-pruned SNP set was generated from the NHGRI list using r^2^<0.3. We then performed an eQTL enrichment analysis on this set, using a permutation procedure on 100 randomized sets of SNPs that reflect the LD-pruned set's MAF distribution. To show that the eQTL enrichment is not dependent on the particular LD-pruned set chosen, we generated 100 such LD-pruned sets on each of which 100 simulations were performed, as just described (Figure S3).

### Master Regulator Enrichment Analysis

A similar enrichment analysis was simultaneously conducted on the number of master regulators, defined as eQTLs which control the expression of at least 10 genes at a given expression p-value. As before, this analysis was done across a range of p-values thresholds (10^−8^ to 10^−4^) to establish robustness. We also investigated whether the enrichment of master regulators or eQTL hot spots in the NHGRI list depends crucially on the number of target genes (10, 50, 100, 1000) in the definition (Figure S4).

### Assessing Robustness across Phenotype

It is widely appreciated that LCLs are not likely to be faithful models for gene expression in all tissues. To assess whether our findings for the associated SNPs from the NHGRI catalog were driven by enrichment in a few of the diseases for which LCLs might be reasonable proxies for tissues most strongly implicated in disease, we examined separately results for all disorders for which brain is the likely focus of disease (neurological and psychiatric disorders), all cancer phenotypes, since it might be hypothesized that the most relevant tissue was the site of the original tumor, and autoimmune disorders for which LCLs are, arguably, a reasonable proxy for tissue relevant to the disease process. Results are summarized in Table 1. We also tested whether the level of enrichment for eQTLs (defined using p<10^−6^ in studies on the CEU) in SNPs identified in studies on autoimmune disorders was significantly different from the level of enrichment for eQTLs in SNPs identified in studies of all other disorders using a chi-square test with 1 df.

### Expression Score for Annotating SNPs

Given the results of our studies on the SNPs reproducibly associated with complex traits, we reasoned that prioritizing follow up of SNPs discovered to show phenotype associations through GWAS should be improved by adding eQTL information to the physical and publicly available functional information commonly used for SNP annotation. Thus, we constructed a score for each SNP that quantifies the likelihood that the SNP has a function (or in strong LD with a functional SNP) in regulating transcript levels. Using the SCAN db file that has annotation information for each SNP (location, host gene, function, CEU MAF) as well as summaries of eQTL p-values (minimum cis p-value, minimum overall p-value), we focused on the 3,844,039 unique autosomal SNPs. The eQTL p-values were truncated at 0.01 for cis-regulatory SNPs (360,696 SNPs with p-value<0.01) and 0.0001 for trans-regulatory SNPs (1,883,759 SNPs). We define a cis-regulatory SNP as a SNP that is within 4 MB of the beginning or end of the gene with which the SNP shows transcript level association, and we define a trans-regulatory SNP as a SNP that is >4 MB from (or on another chromosome than) any gene with which the SNP shows transcript level association.

In order to devise a one-dimensional score that summarizes as much of the eQTL signal information as possible for prioritizing GWAS signals according to their “functionality”, we first note that neither the scale of the score nor the distribution of the score for non-functional SNPs are relevant – just the appropriate ranking of SNPs with a function in regulating transcript levels. The building blocks for the SNP score are: p_o_ which is the overall smallest eQTL p-value (it can be either cis or trans), p_c_ which is the smallest cis p-value, N_o_ which is the overall number of transcripts used in the analysis, and N_c_ which is the number of cis transcripts (i.e. the number of transcripts annotated to genes in the vicinity of the SNP under investigation). The score we propose:
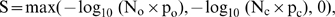
builds on Bonferroni corrections applied separately for trans and cis signals (there are 13,080 transcripts used in the analysis). Note that the scores are truncated at zero.

### Utilizing Expression Information in GWAS

In order to determine whether our newly derived functional score can aid in identifying associations likely to be replicated, we first examined the summary data for Crohn's disease from the WTCCC association study [11] (downloaded when results data were publicly available). After QC (less than 5% missing data, good clustering), we filtered SNPs at a 1% MAF threshold, and removed markers with no rs ID, leaving 391,878 SNPs. We matched these SNPs using the rs IDs within the SCAN database, leaving us with 386,306 SNPs.

We then ordered the SNPs according to their score *S* from largest to smallest score. The top SNPs based on the eQTL score show an excess of small association p-values with Crohn's disease (for example, in the top ten SNPs, six have p-values smaller than 0.01).

We divided the SNPs into groups of 10,000 (in the order of their eQTL scores) and counted the number of SNPs in each group with p<0.01 for association with Crohn's disease - see Figure 2 (there are just over 123K SNPs with scores larger than 0). The first 10,000 SNPs (ranked according to eQTL function score) include 357 with Crohn's association p-values smaller than 0.01. We simulated sets of SNPs with allele frequencies matching the top 10,000 and, in 100 simulations, the range of this statistic (number of p-values smaller than 0.01) is 117–178 which is far less than the observed number.

We also examined enrichment for eQTLs in the Crohn's disease data by ordering the SNPs showing association to Crohn's, and examined the proportion of SNPs with eQTL scores >3, >2, and >1 among the top 1000 SNPs for Crohn's disease (Figure 3).

We repeated the Crohn's disease enrichment studies in the other WTCCC phenotypes. As with Crohn's disease, we first conducted analyses checking for an excess of small association p-values in the SNPs that have high expression annotation scores (Figure 4). Finally, we ranked the SNPs according to their association p-values and looked for an enrichment of high expression scores (Figure 5).

To insure that results of enrichment studies were not unduly influenced by large numbers of correlated SNPs in the MHC region with high eQTL scores, we repeated the analyses summarized in Figure 2 and Figure 4 after removing all SNPs on chromosome 6 (see Figure S5). Results were relatively unchanged.

## Supporting Information

Figure S1 Minor allele frequency (MAF) distributions for Affymetrix 6.0 SNPs (A), Illumina 1M SNPs (B), and NHGRI Associated SNPs (C).
(1.57 MB TIF)

Figure S2 (A) The distribution of the number of eQTLs (at p<10^−4^ observed for each of 1,000 draws of 1,598 SNPs from bins matched for minor allele frequency to the 1,598 SNPs downloaded from the NHGRI catalog (bins include all HapMap SNPs) is shown in the bar graphs, with the actual number of eQTLs observed in the 1,598 SNPs from the NHGRI catalog shown as a solid circle. (B) Identical to Figure 1, leftmost panel, with analysis as above, except that SNPs for the simulation were drawn from SNPs on high-throughput GWAS panels.
(2.69 MB TIF)

Figure S3 Observed numbers of eQTLs among all trait-associated SNPs trimmed for LD are shown as solid black circles with the bar graph showing the distribution of eQTLs among SNPs chosen at random from the same allele frequency bins from among all SNPs included on high-throughput GWAS products. Enrichment of eQTLs among trait-associated SNPs is preserved even in the absence of LD among trait-associated SNPs.
(2.09 MB TIF)

Figure S4 Observed numbers of master regulators among all trait-associated SNPs are indicated by the solid black circles and the distribution of the number of master regulators (defined as SNPs predicting 10 transcripts and SNPs predicting 100 transcripts, both for p<10^−4^) observed in 1,000 draws of 1,598 SNPs from minor-allele-frequency-matched bins (including all SNPs on Illumina 1M and Affymetrix 6.0 products) is plotted in the bar graphs.
(2.14 MB TIF)

Figure S5 This is similar to Figure 2, except that all SNPs on chromosome 6 have been removed from calculations, demonstrating that the Crohn's enrichment for eQTLs is not dependent on SNPs in the HLA region. Results were similar for T1D and RA.
(0.88 MB TIF)
