# Supplementary figures and images for: Trait-Associated SNPs Are More Likely to Be eQTLs: Annotation to Enhance Discovery from GWAS
Source: PLoS Genet. 2010 Apr 1;6(4):e1000888. doi: 10.1371/journal.pgen.1000888 (PMC2848547; doi:10.1371/journal.pgen.1000888)

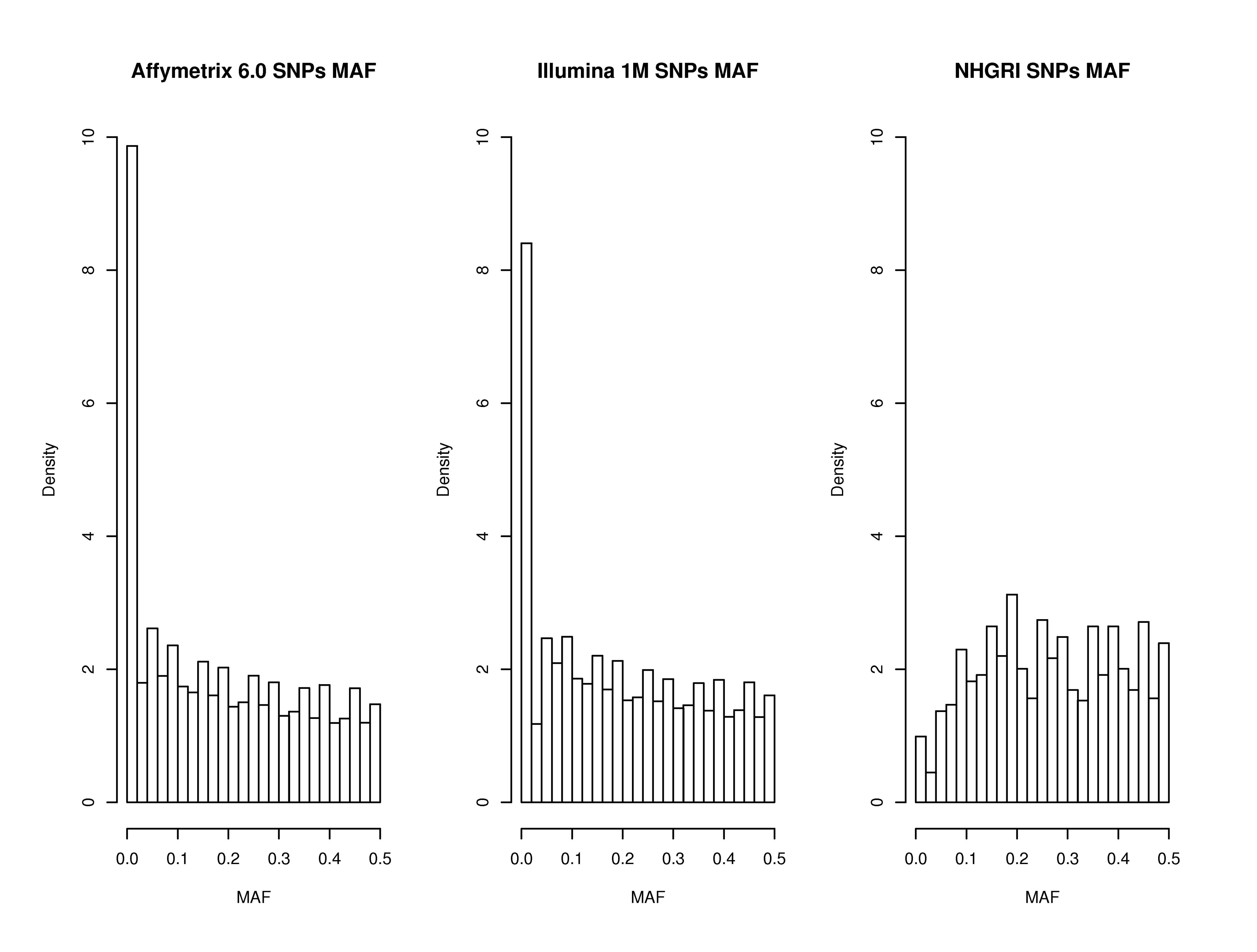

Supplement: Figure S1 — Minor allele frequency (MAF) distributions for Affymetrix 6.0 SNPs (A), Illumina 1M SNPs (B), and NHGRI Associated SNPs (C). (1.57 MB TIF) [file pgen.1000888.s001.tif]

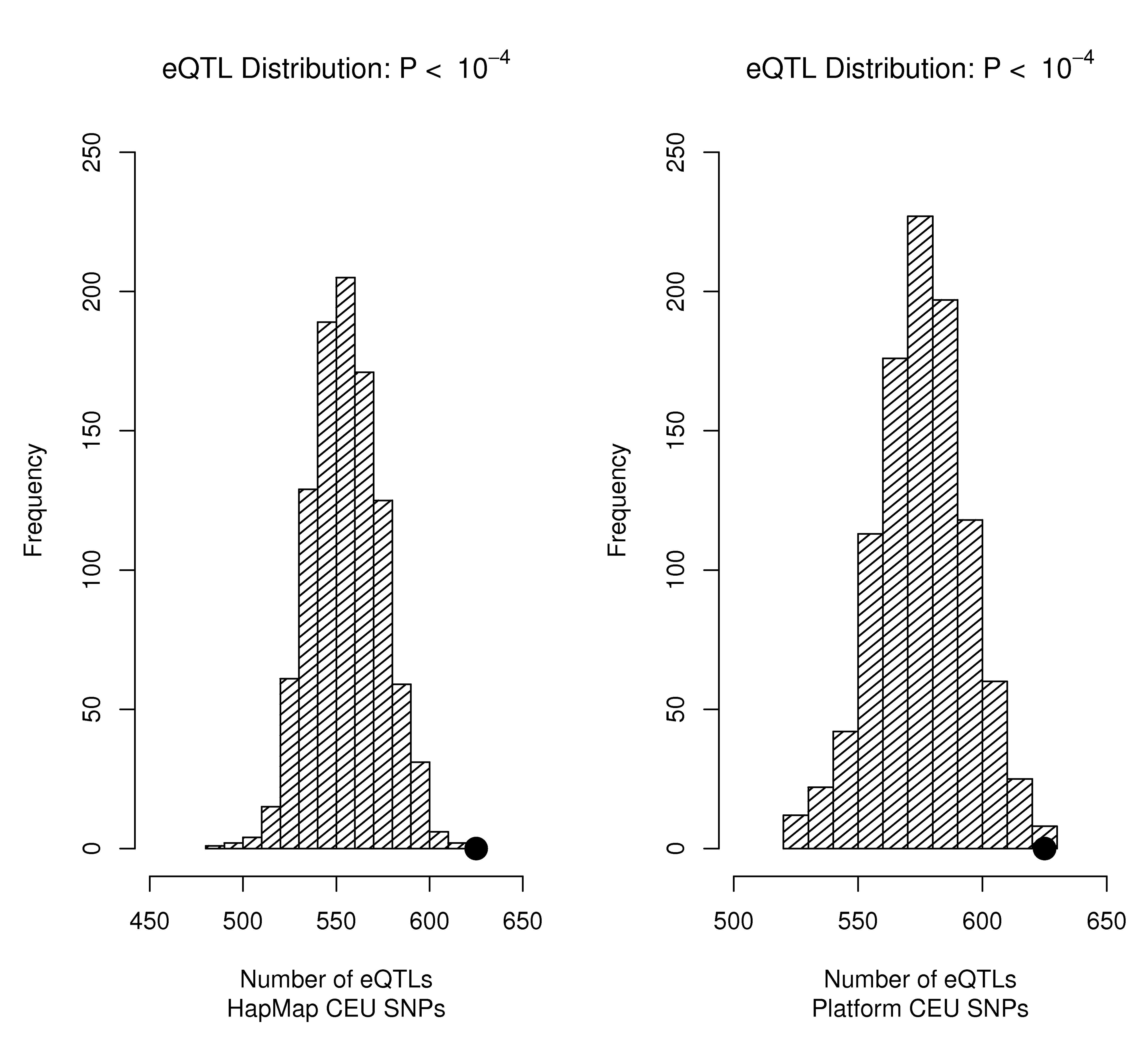

Supplement: Figure S2 — (A) The distribution of the number of eQTLs (at p<10−4 observed for each of 1,000 draws of 1,598 SNPs from bins matched for minor allele frequency to the 1,598 SNPs downloaded from the NHGRI catalog (bins include all HapMap SNPs) is shown in the bar graphs, with the actual number of eQTLs observed in the 1,598 SNPs from the NHGRI catalog shown as a solid circle. (B) Identical to Figure 1, leftmost panel, with analysis as above, except that SNPs for the simulation were drawn from SNPs on high-throughput GWAS panels. (2.69 MB TIF) [file pgen.1000888.s002.tif]

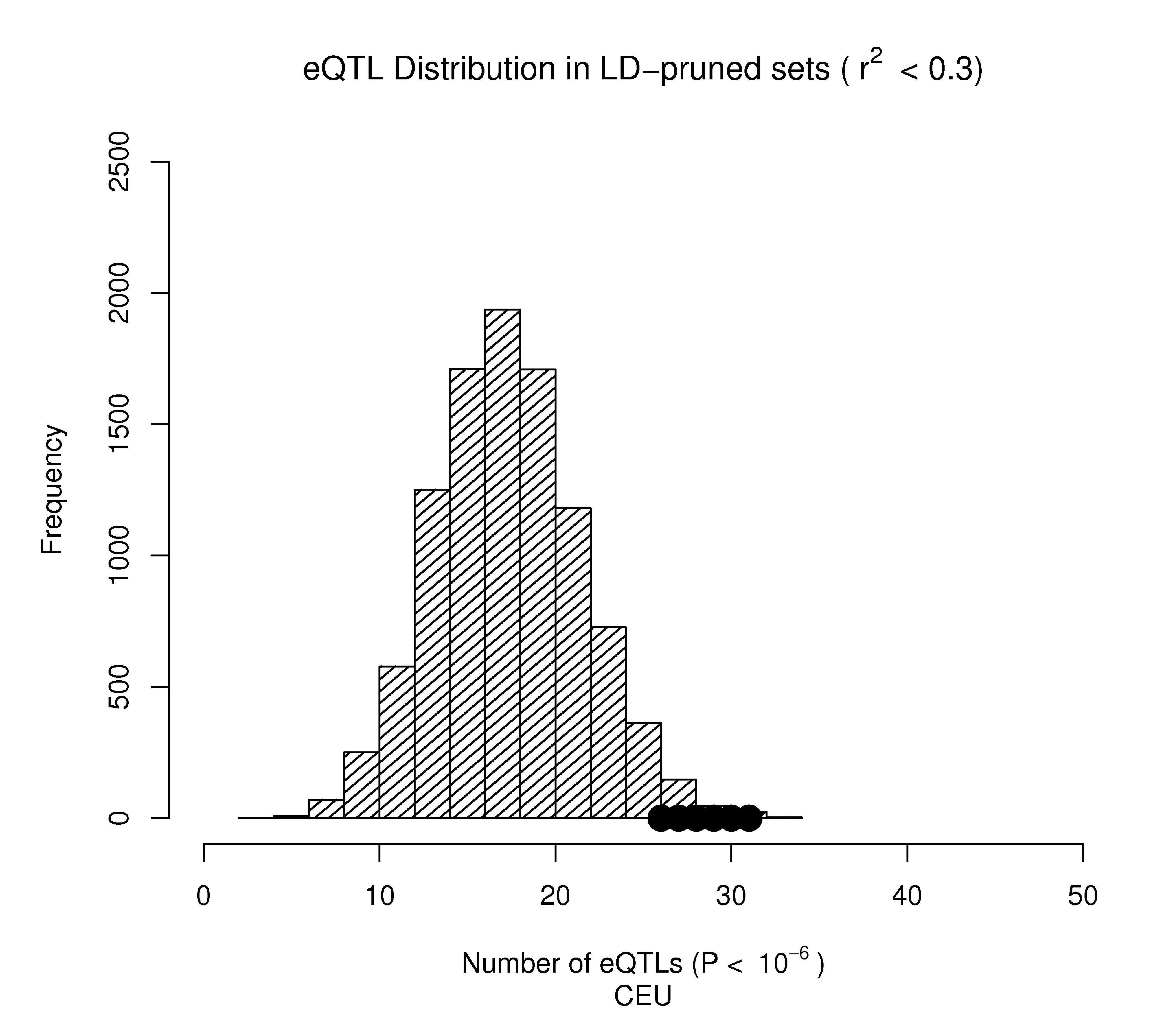

Supplement: Figure S3 — Observed numbers of eQTLs among all trait-associated SNPs trimmed for LD are shown as solid black circles with the bar graph showing the distribution of eQTLs among SNPs chosen at random from the same allele frequency bins from among all SNPs included on high-throughput GWAS products. Enrichment of eQTLs among trait-associated SNPs is preserved even in the absence of LD among trait-associated SNPs. (2.09 MB TIF) [file pgen.1000888.s003.tif]

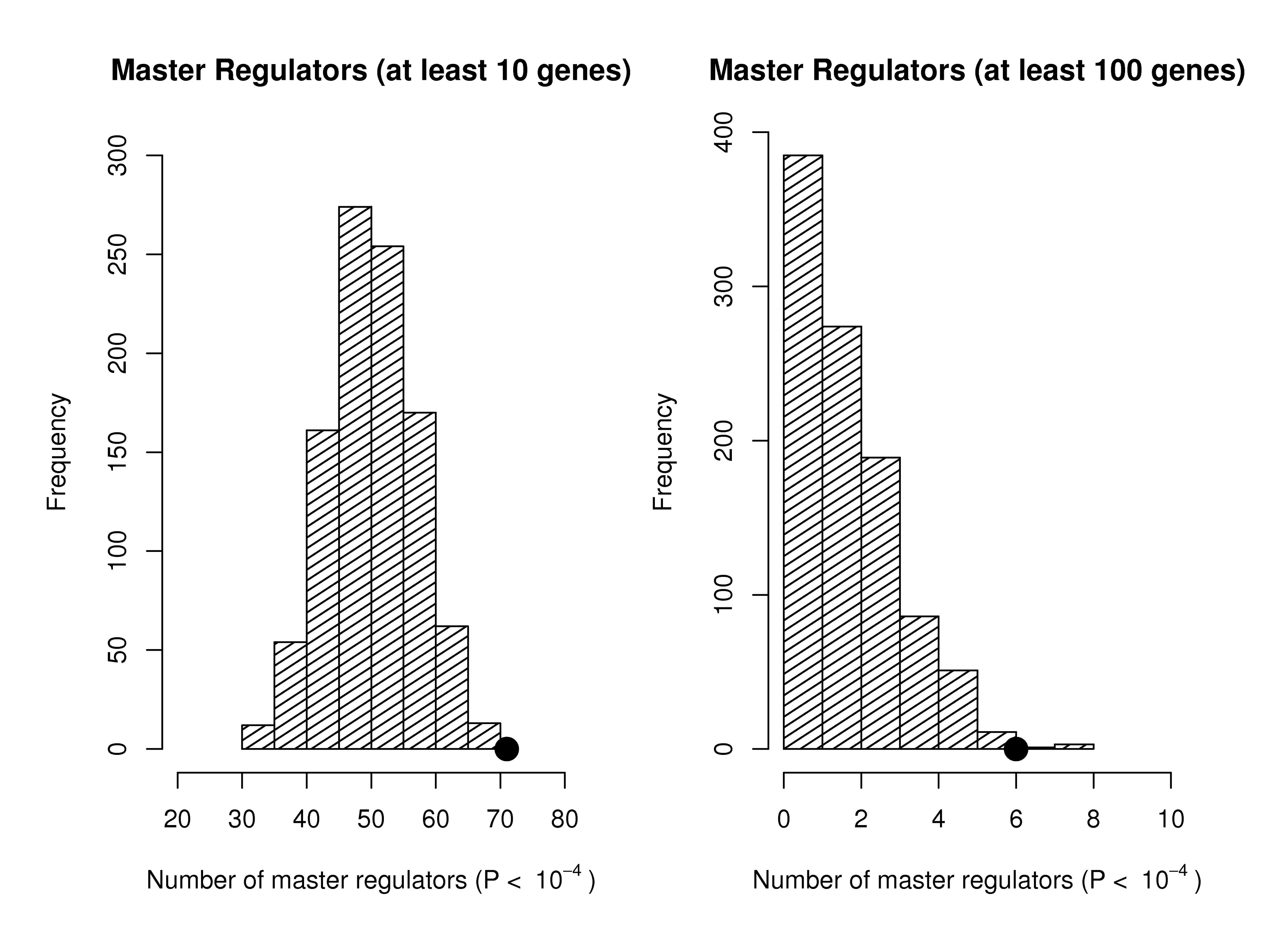

Supplement: Figure S4 — Observed numbers of master regulators among all trait-associated SNPs are indicated by the solid black circles and the distribution of the number of master regulators (defined as SNPs predicting 10 transcripts and SNPs predicting 100 transcripts, both for p<10−4) observed in 1,000 draws of 1,598 SNPs from minor-allele-frequency-matched bins (including all SNPs on Illumina 1M and Affymetrix 6.0 products) is plotted in the bar graphs. (2.14 MB TIF) [file pgen.1000888.s004.tif]

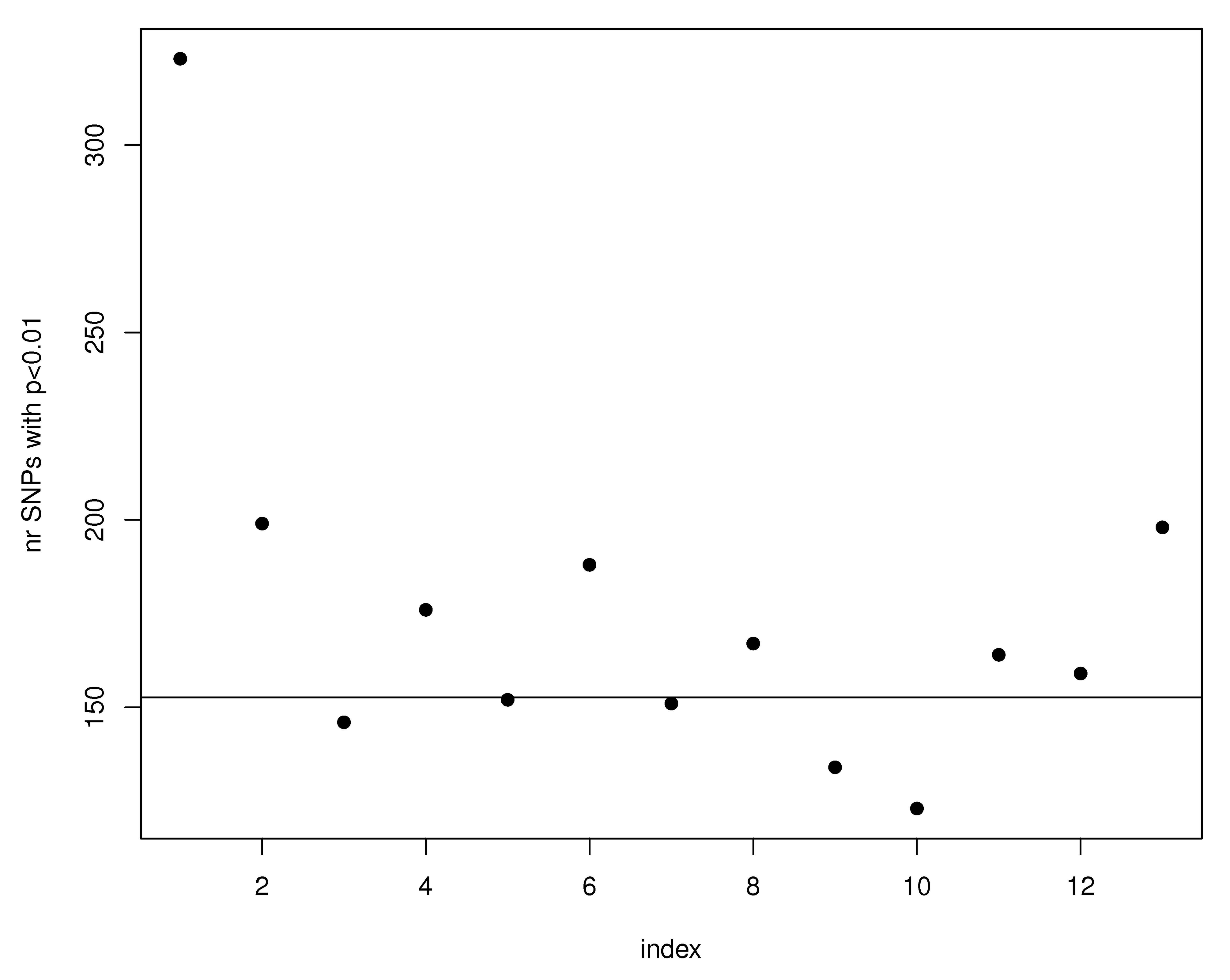

Supplement: Figure S5 — This is similar to Figure 2, except that all SNPs on chromosome 6 have been removed from calculations, demonstrating that the Crohn's enrichment for eQTLs is not dependent on SNPs in the HLA region. Results were similar for T1D and RA. (0.88 MB TIF) [file pgen.1000888.s005.tif]
